# Managerial Responses to the Onset of the COVID-19 Pandemic in Healthcare Organizations Project Management

**DOI:** 10.3390/ijerph182212082

**Published:** 2021-11-17

**Authors:** Ariadna Linda Bednarz, Marta Borkowska-Bierć, Marek Matejun

**Affiliations:** 1 Department of Management and Logistics in Health Care, Medical University of Lodz, 90-131 Lodz, Poland; marta.borkowskabierc@gmail.com; 2 Military Institute of Medicine, 04-141 Warsaw, Poland; 3 Department of Entrepreneurship and Industrial Policy, Faculty of Management, University of Lodz, 90-237 Lodz, Poland; marek.matejun@uni.lodz.pl

**Keywords:** project management, COVID-19 pandemic, healthcare management, risk analysis, project performance

## Abstract

The goal of this study was to identify and assess the impact of the COVID-19 pandemic on project management practices in healthcare organizations, taking into account, in particular, risk analysis, project performance, organization of the work of project teams, and tendencies in future developments in project management. In order to achieve this goal, a study was conducted on 20 project managers in selected healthcare organizations in Poland. The results indicate that a systematically conducted risk analysis as part of the implemented projects enabled rapid and effective reactions during the COVID-19 pandemic. The pandemic has also significantly altered the organization of the work of project teams and, in the opinion of the research subjects, this will significantly impact future solutions for project management in healthcare organizations. The obtained results form a basis for the increase in professionalization in project management in healthcare organizations.

## 1. Introduction

The COVID-19 pandemic, the genesis of which dates to early December 2019 in Wuhan, Hubei Province, China [1], has resulted in a series of unprecedented global economic, social, political and cultural implications [2,3,4,5]. The pandemic period has also been characterized by intensified implementation of many projects within healthcare organizations, not only in the areas of medical research, new solutions for work, telemedicine and IT, but also in the organization of the medical services system and of infrastructural solutions. The sudden and dynamically changing situation has left little time for complex planning and for effective implementation, thus creating challenging conditions for the implementation of projects while requiring highest competency levels on the part of managers. These conditions were also determined by the nature of healthcare organizations’ operations and by the diversification of their stakeholders, requiring a high level of professionalism in project management.

Despite many analyses concerning changes in project management in various industries (mainly relating to IT, e-commerce, R&D and heavy industries) there are no detailed, in-depth results concerning managerial responses to the COVID-19 pandemic onset in healthcare organizations project management. This was noted, in particular, by P. Jiang et al. [6], who identified a significant research gap concerning the in-depth studies on the multidimensional impact of the COVID-19 pandemic on healthcare and on the orientation of sustainable future planning in this sector. Taking this into account, the goal of this study was the identification and assessment of the COVID-19 pandemic’s impact on practices of project management in healthcare organizations, taking into account, in particular, the area of risk analysis, project performance and organization of the work of project teams. Taking the forecasts of the global future impact of the pandemic into account [7,8,9], this goal was expanded by indicating the potential directions of the pandemic’s impact on the prospects of project management in healthcare organizations.

In order to achieve this goal, this study was conducted on 20 project managers in selected healthcare organizations in Poland. The first part of this paper presents a literature review report, and uses it to formulate four research questions. Then, materials and methods are discussed, taking into account, in particular, the description of the sample and the research tool, and the characteristics of the respondents. The next section presents the results of the study, divided into four parts concerning: (1) the preparation of healthcare organizations in the aspects of risk management in projects, and the assessment of the COVID-19 pandemic’s impact on (2) the effectiveness criteria of implemented projects, (3) the organization of the work of project teams, and (4) the future of project management in healthcare organizations. As part of the scientific discussion, answers are provided to the posed research questions, the obtained results are confronted with the results of the previous studies, and the limitations of the research procedure are also discussed. The summary highlights the main scientific contribution of the work, formulates practical implications, and establishes long-term directions of future research in project management in healthcare organizations.

## 2. Literature Review and Research Questions

The concept of the project—even though it is relatively recent in its current form—was known over 4 thousand years ago. It is assumed that one of the first implemented projects were the well-known monuments to civilization, such as the Egyptian pyramids in Giza (approx. 2570 BCE), the statue of Zeus at Olympia (approx. 430 BCE) or the Great Wall of China (approx. 210 BCE) [10]. The first modern enterprise which used structured management techniques and methods is considered to be the “Manhattan” project—an initiative of the United States government in order to construct the atomic bomb in the early 1940s. Even though the intricate details of those methods were treated as a military secret, this knowledge reached the business world and started to be also used in commercial activities [11]. In 1996, a series of handbooks known as “system program management” were published, forming a basis for the field’s dynamic development not only in the area of defense, but also in government, social and business projects [12] (p. 530).

The project management methodologies and standards commercially available today have different definitions of a project [13] (p. 13), [14] (p. 4), [15] (p. 288). Regarding the literature definitions, a project can be anything that is created from scratch or a significant modification to an existing system, which requires substantial effort in terms of development and delivery. In this context a project has a specific product (output), objectives (outcome), defined timeline (schedule), budget, and various other parameters (resources, quality, risks, etc.) [16] (p. 13). In his definition, J.R. Turner [17] (p. 2) points out these different aspects and constraints of a project: “A project is an endeavor in which human, financial, and material resources are organized in a novel way to undertake a unique scope of work, of given specification, within constraints of cost and time, so as to achieve beneficial change defined by quantitative and qualitative objectives”.

By reviewing the definitions, it can thus be established that every project is characterized by specific features. Most approaches (both those quoted above and other approaches in scientific literature) are based on three aspects: scope, time and budget.

These areas may be subject to measurement and assessment, which express the performance of specific projects. For example, the PRINCE2 (Projects IN Controlled Environments) methodology defines six dimensions of project management performance: time, cost, quality, scope, risk and benefits [18]. Although these are the basic attributes of each project, one should not forget other areas which require effective management, such as project structure, human resources, communication or supply [19]. Project success frequently depends on the combination of goals and their skillful management in the perspective of the organization (benefits), in addition to the customer (satisfaction) or project participant (professional development, stakeholders’ goals) [20,21].

Historically, the manner of cooperation has changed, from individualized work to teamwork and joint problem-solving, which fosters more effective use of knowledge and more dynamic processes [22]. It may be assumed that the pace of change in modern world generates a growing need for innovative solutions, and thus, the number of successfully implemented projects [23] (p. 5). In light of such an argument, it seems justified to consider the skill of project management to be a strategic strength of an organization, which enables a more effective implementation of a company’s strategy, while outdistancing the competition [24] (pp. 14–15). The organization’s ability to manage projects may be simultaneously measured, assessed and expressed in the form of a specific level of project maturity [25,26].

Although each project is undertaken in order to achieve success, as many as 19% (in the case of IT) have ended in failure, and 50% had significant problems with project completion, leaving only 31% of the analyzed initiatives achieving that planned results within the planned deadlines and budget [27].

An in-depth analysis has demonstrated that the healthcare sector does not differ in statistics from other sectors of the economy. However, along with the development of project management techniques, an increase in projects designated “successful” can be seen, from 16% in 1994 to 35% on average in the years 2015–2020.

There are many reasons for project failure identified in the literature. According to F. Liebert [28], the key barriers to project management include: (1) problems and issues associated with organizational culture; (2) the inability to properly manage risk in an IT project; and (3) lack of knowledge of development team members. A. Lee, K. Moon and S. Kim [29], based on a 2010–2018 literature review, identified 135 causes of project failure. They included among the key barriers: establishing of (1) unclear project plans; (2) unclear project goals and (3) unclear requirements related mainly to not understanding or not accepting fully the expectations of the end user. Further studies utilizing factor analysis have distinguished 10 categories of project management barriers, which included aspects such as difficulty of process management, stakeholder conflict or disturbance of communication. Factors which may influence the success or failure of a project also include clear division of roles and responsibilities within a team, ongoing monitoring of actions and verification of milestones [30], in addition to the development of appropriate skills by managers and members of project teams, i.e., technical, leadership, business and digital skills [31].

Effective project management generates both qualitative and quantitative benefits [32]. It becomes a tool for change management [33] and implementation of complex initiatives which require interdisciplinary approaches [34]. Therefore, the development of professionalization in the form of starting project management offices, implementing agile project management methodologies, and improving an organization’s existing processes and personnel to support project activity is not surprising. Studies conducted in Australia [35] demonstrate that 64% percent of survey respondents think that the complexity of projects has increased over the past decade and project management skills will be more important in the future.

The need for professionalization of managerial competences can also be seen in the healthcare sector [36,37], in addition to the progressing awareness of the need for effective project management [38]. For a long time, hospital managers did not notice the potential resulting from the implementation of project management methods due to the operational specifics of their facilities. Furthermore, hospitals frequently encounter project activities as part of their daily activities, e.g., prevention programs, IT or education projects, research initiatives including clinical trials, or organizational change projects. The healthcare sector is also an area where a dynamic development of IT technologies is underway, such as using artificial intelligence for diagnostic purposes [39].

The need for professionalization in the area of project management is also increased by the complexity and the number of variables impacting medical entities [40]. This is a sector that is characterized by relationships with multiple stakeholders, overlapping goals of activity, complicated decision-making systems and autonomy of facilities [41]. The buyer–product–seller relationship present in other industries in the case of healthcare must include other actors in the form of medical personnel (independent in their treatment decisions), insurer, payer, local government or other funding and supervising entity. A project manager in the healthcare sector should understand the dependencies present within it and should manage the project in a manner which accounts for the potential impact on and by all stakeholders. Achieving this is facilitated by the use of clear standards offered by project management methodologies. Organizations which are capable of rapidly adapting to the changes occurring in the environment by operating based on agile methodologies deal better with the current crisis than companies operating based on traditional patterns [42]. The use of agile project management methodologies also alleviates feelings of emotional exhaustion resulting from the pressure of work on project teams during the COVID-19 pandemic [43] and supports the development of Industry 4.0, and thus Healthcare 4.0 [44]. In the healthcare sector agility, plays a vital role in COVID-19 care to enhance the operational performance to meet the dynamic demand of COVID-19 hospitals [45].

Risk management is a key area of project management and healthcare [46]. Each new undertaking should be preceded by a thorough analysis of potential risks, taking both hazards and a project’s chances of success into account [47]. It is impossible to eliminate all risks, so it is necessary to identify and manage the most important of them in order to avoid project failure [48]. However, in an attempt to minimize the number of risks and failures, in-depth investigation is often applied, which in consequence increases system complexity and raises the possibility of other risks or of occurrence of unexpected events [49]. This phenomenon has led to the development of resilient health care (RHC), which views humans as a positive resource for coping with disturbances in complex systems [50,51]. Resilience is then seen as a capacity for flexibility, robustness and adaptability in response to changing circumstances, minimizing the impact on an entity’s performance and safety [52]. The concept aims at “deliberate design and construction of systems that have the capacity of resilience” [53] (p. 381) by creating conditions that support resilient performance. E. Hollnagel [54] suggests focusing on four potentials: anticipation, monitoring, responding and learning, and T.A. Saurin et al. compiled six guidelines to achieve this: (1) give visibility to processes and outcomes; (2) monitor unintended consequences of improvements and small changes; (3) encourage diversity of perspectives when making decisions; (4) design slack; (5) monitor and understand the gap between prescription and practice; and (6) create an environment that supports resilience [55,56,57]. The resilient engineering aspect has been also noticed in project management [58], and has criticized modern project risk management practices for being time-consuming and inflexible when acting in a crisis or a rapidly changing environment [59]. As academic research is focusing on the concept of resilience in project management, it is also being partly introduced in the commonly used project management methodologies as a part of risk management in the context of emergent risk.

During 2020, project managers were confronted with more risks related to the consequences of the COVID-19 pandemic outbreak [60]. Although no project is likely to have accounted for such a widespread pandemic in its risk analysis, some elements related to the pandemic may have already been present. A key difficulty in this case was the sudden materialization of multiple risks at the same time. The deployment of risk management in healthcare has traditionally focused on patient safety and on ensuring an organization’s ability to achieve its mission and financial performance. In recent years, a clear shift in risk management towards composite proactive risk analyses [61], in addition to the perception of risk through the much broader lens of the entire healthcare system performance and resilience [62], can be seen. This therefore leads to formulating the first research question:

RQ1: Have risk analyses in project management in healthcare organizations enabled a rapid and effective reaction due to the risk that materialized during the COVID-19 pandemic?

The dynamic and unexpected materialization of multiple risks related to the COVID-19 pandemic may, in many cases, have a negative impact on the performance of the implemented projects. This can be seen, in particular, in the construction industry [63,64,65], where the COVID-19 pandemic had a negative impact on the timely implementation of construction projects. The results of other research indicate that the pandemic also had a negative impact of the performance of projects in the energy sector [66], infrastructure [67] or crowd-funding projects [68].

The results of an in-depth qualitative study conducted by A. Alsharef et al. [69] identify 11 symptoms of the pandemic’s negative impact on project performance through: (1) disparities among the approaches of state authorities; (2) material delivery delays and shortages; (3) delays in inspections and securing of permits; (4) reduction in the efficiency and productivity rates; (5) suspension or slowing of ongoing projects and delay in the start of new ones; (6) price escalations and additional, unexpected costs; (7) safety concerns; (8) increased number of disputes, litigations and claims by external stakeholders; (9) workforce-related challenges; (10) increase in demand from local suppliers; and (11) transition to work from home. Simultaneously, the respondents identified specific new, advantageous opportunities resulting from the COVID-19 pandemic, such as: (1) the ability to secure loans at low interest rates; (2) increase in demand for specific types of projects, e.g., transportation, residential, and medical; (3) possibility of recruiting skilled workers, who are being let go from competitors; and (4) more time for conducting internal reviews and improving existing project management systems.

The literature also presents good practices for the performance of healthcare sector-related projects under the conditions of the COVID-19 pandemic. Examples include the Iranian Red Crescent project of launching testing centers for the coronavirus disease [70], or the UK Ventilator Challenge project, which engaged over 50 companies across diverse sectors ranging from medical devices to the automotive, aerospace and defense industries [71]. Although these projects ended with success, the authors point out that further studies are needed in order to analyze costs and long-term benefits resulting from their performance. This leads, therefore, to formulating the second research question:

RQ2: In what manner has the COVID-19 pandemic impacted project management performance in healthcare organizations?

The introduction during the COVID-19 pandemic, almost everywhere in the world, of large-scale interventions, such as physical distancing or lockdowns [72], has resulted in organizations and project teams having to adapt to the new situation. As an effect, an unprecedented shift from offline to online activities resulting in an accelerated use of digital technologies was observed [73,74]. Even though the development of areas of e-commerce, e-health and e-government was previously observed for a number years, the introduced lockdowns provided the final stimulus and motivation for more rapid implementations. For example, an e-commerce branch noted a significant increase in the sales volume during lockdown [75,76], which translated into the initiation of subsequent projects which use state of the art achievements in technology and artificial intelligence [77].

Significant changes were also observed in the organization of the work of project teams. Examples include experiences from the performance of a R&D project during the COVID-19 pandemic analyzed by W.L.M de Mendonça et al. [78]. In-depth qualitative studies mainly demonstrated the transition from work on-site to work from home, the introduction of remote communication, changes to the manner of assigning tasks and of handing the requirements over to the developers, and changes in relations with project stakeholders due to the reduction in the frequency of meetings.

Similar changes were indicated by the results of a study by S. Telin and N. Esmail [79]. A significant expansion of digital communication was observed, with attention being paid to two significant aspects resulting from the specifics of remote work: accessibility to office instruments in the remote environments, and ensuring the physical and mental health of the members of project teams. Changes have also included challenges in the sphere of leadership related to maintaining the organizational culture and ensuring that the employees experience the culture in a remote work environment [80], and motivating and building engagement in the remote workplace [81]. In these conditions, leadership plays a significant role, in the form of supervisory support resources, which increases engagement, indirectly contributing to the increase in adaptability and proactivity of the project team [82].

Like in other sectors of economy, changes in the organization of project teams towards the virtualization of activity and remote work while ensuring maximum safety at the workplace have also occurred in healthcare organizations [83]. Healthcare systems can build their resilience in order to cope with complexity and various adverse events by adapting H4.0 (Healthcare 4.0). This enables customization using principles and applications from Industry 4.0 (meaning a set of “digital information and communication technologies aimed at promoting higher levels of automation and interconnectivity”) [84]. According to an assessment by G. Tortorella et al., the highest overall impact is provided by: (1) remote consultations and development of plan of care; (2) digital non-invasive care; (3) interconnected medical emergency support; and (4) digital platforms for collaborative sharing of patients’ data and information. As each hospital has its specific characteristics and level of technological development, these technologies can boost their resilience by reducing the reliance on human adaptive skills—especially during a COVID-19 outbreak. H4.0 may support healthcare organizations in these unprecedented times in terms of general management, i.e., data access, team leading, or knowledge and change management.

Moreover, due to the pandemic, a change in priorities of the activities of research teams was observed [85]. This, therefore, leads to formulating the third research question:

RQ3: How did the COVID-19 pandemic influence the organization of work of project teams in healthcare organizations?

The significant and multidimensional impact of the COVID-19 pandemic on managerial practices, also in the healthcare system, forms the basis for considerations of the durability and future of these changes. R. Assaad and I.H. El-adaway [86] point out specific directions of the pandemic’s impact on the future functioning of organizations concerning workforce-related issues, procurement and supply chain implications, contractual, legal, and insurance aspects, and project management practices and the workplace environment.

R. Müller and G. Klein [87] forecast an increased interest in risk in project management. In their opinion it will be important to understand the relationships between various types of risk and the introduction of new solutions in project management (e.g., development of new values and priorities), which will increase the resilience to crises of organizations and implemented projects. The future challenges they identified also include changes in leadership and management styles which result from the virtualization and digitization [88] of the work of project team members.

In this context, T. Wu [89] anticipates an increasing digitization of project management, whereas P. Sonjit, N. Dacre and D. Baxter [90] anticipate a dynamic development of homeworking project management, which is related to, among other factors, preparing a technological infrastructure that provides project team members equal access to information and enables them to perform specific tasks from home. Predictions concerning the long-term effects of the pandemic also indicate the consolidation and progressive development of remote solutions, i.e., remote work, telework [91,92] or hybrid solutions [93]. Multiple analyses indicate both the positive and negative effects of such solutions [94,95,96,97].

Anticipated changes in project management also present a very significant challenge to the healthcare industry, which had already long been subject to a digital revolution driven by big data, machine learning and artificial intelligence. The pandemic increased the need for investment in robust health data infrastructures and pipelines to minimize or eliminate barriers and latency to gather, assimilate, validate and share data widely and swiftly [98]. This, therefore, leads to formulating the fourth research question:

RQ4: How will the COVID-19 pandemic impact the future of project management in healthcare organizations?

In order to answer the research questions posed above, an empirical study was conducted, the results of which are presented below.

## 3. Materials and Methods

### 3.1. Sample and Research Tool

The study was conducted on 20 representatives (respondents) of the healthcare sector organizations (hospitals) on at least the 2nd reference level according to the Ministry of Health in Poland. These hospitals provide health services in at least nine medical specialties and usually are engaged in larger, more complex and interdisciplinary projects than hospitals on the 1st reference level. The sample of respondents was selected among 60 managers taking part in the MBA (Master of Business Administration) in Healthcare Organizations studies carried out at the request of the Polish Ministry of Health. All these managers represented hospitals on at least the 2nd reference level. The selection of respondents for the sample was purposive and was conducted in 2 stages:

- Stage 1: Due to the subject matter of the study, the selection focused on respondents holding managerial positions in projects carried out in hospitals or supporting the implementation of projects from a level of functional manager/representative of the management board supervising the implementation of key projects in the organization. Among 60 participants of the MBA studies, 37 met this criterion.
- Stage 2: For more valuable and in-depth results, selection focused on respondents from hospitals with a larger number of projects (at least 10 implemented during a year) and their significant variation (at least three kinds from a list of research, infrastructural, IT, organizational change, clinical trials, and preventive or educational projects). On the basis of preliminary interviews among 37 participants of the MBA studies, 26 met this criterion.

Finally, among the 26 managers, 20 agreed to participate in research. The study was conducted in March of 2021 using a questionnaire-based interview method, in the form of individual, personal meetings.

In the research process, special attention was paid to ethical issues [99], which included obtaining free, informed consent for participation in the study from the respondents, and ensuring the anonymity of the collected empirical material and the respondent’s right to withdraw from the study at any stage of the research process. Moreover, the respondents agreed for their statements to be quoted in academic publications, on the condition that the statement is presented in an anonymous manner.

The questionnaire used in the study consisted of three parts (the full contents of the questionnaire constitutes Appendix A): Part 1: “Respondent’s data”—gender, managerial experience and role, type and nature of the implemented projects. Part 2: “General characteristics of project management in the organization”—the operational range of the organization, assessment of the organization’s risk and project management maturity level, assessment of the difficulty/complexity and innovation of the implemented initiatives. Part 3: “Managing projects during the COVID-19 pandemic”—assessment of: the organization’s pandemic preparedness from the point of view of project risk management; the pandemic’s impact on the performance of commenced projects; most important changes and challenges in the area of project team work organization during the pandemic; the impact of the pandemic on the manner of project management in healthcare organizations in the future.

In evaluating the questions, a 5-point Likert scale was used. The interview concentrated on open questions concerning the experiences, feelings and remarks of the respondents on the changes and challenges which occurred in project management under pandemic conditions. The managers’ statements were then subjected to content analysis and grouped into appropriate substantive categories, which enabled providing answers to the posed research questions and to fulfil the goal of the study.

### 3.2. Characteristics of Respondents and Maturity of Project and Risk Management in Selected Healthcare Organizations

A total of 20 respondents participated in the study: 11 women and 9 men, with an average age of 43 years (the youngest: 30 years, the oldest 62 years). All the respondents had a higher education at the master’s level, of which 6 had a doctorate, and one had a postdoctoral degree in medical sciences. Most respondents considered the operational range of their entity to cover all of Poland, compared with 3 representatives of regional hospitals. All the study participants were involved in the performance of projects in their entities on a managerial level. In the analyzed group of managers, eight occupied the highest roles in the team, remaining on the post of a project manager, coordinator or chief investigator, compared to 11 participants who occupied the post of the project’s administrative, technical or functional manager (function used mainly in the case of complicated B&R type projects). One of the respondents acted as a representative of a board of directors, supervising the performance of key projects.

Over half the respondents assessed that they are evenly engaged in both process-oriented projects (“soft” projects) and object-oriented projects (“hard” projects), which proves their experience with the management of various types of projects. The remaining participants in the study almost evenly represent the division into people with experience mainly with hard or with soft projects. Moreover, the participants indicated the types of projects they implemented, demonstrating that the most frequently implemented types of project in the studied group were research and educational projects, and clinical trials (also non-commercial). The least frequent type of project was found to be initiatives concerning IT solutions, although even here 4 respondents declared their engagement in this type of activity.

**Figure 1:**
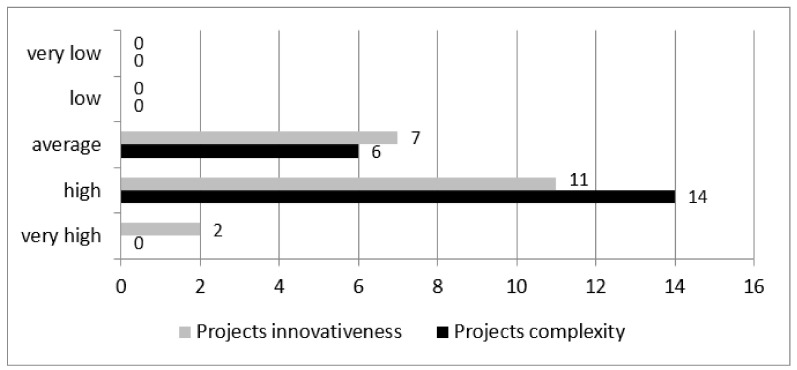
Assessment of the degree of difficulty/complexity and innovation of the performed projects.

The study participants were asked to perform a subjective assessment of the degree of difficulty/complexity and innovation of the performed projects on a 5-point scale: very high, high, average, low, very low (Figure 1).

Most of them assessed the performed initiatives to be difficult and complex, and thus required highly advanced management and administration competencies (14 answers—“high” vs. 6 answers—“average”). At the same time 13 managers assessed their projects to be innovative and very innovative. None of the study participants assessed the aspect of innovation or complexity in their projects at a low or very low level.

**Figure 2:**
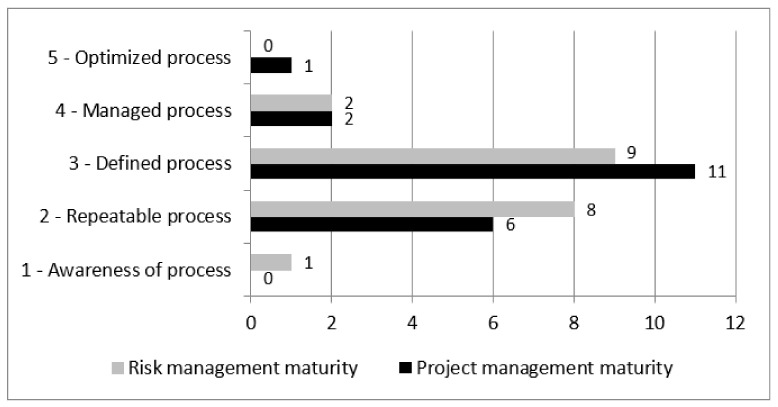
The respondents’ assessment of the project management and risk management maturity levels in their organizations.

The performance of projects—in particular the complex and innovative projects—is never based solely on the competencies of the team or of the project leader. In order to implement such demanding initiatives, an appropriate organization of processes and tools is required, in addition to specific support on the part of the organizational unit in which they are performed. For this reason, the participants were asked questions concerning the organization’s maturity level in the area of project management and the organization’s maturity level in the area of risk management. The P3M3 maturity scale was used for the assessment (Figure 2).

The results in both assessment areas were similar to each other, with a small variation in the extreme values of the assessment. A slightly higher level of maturity can be seen in the project management area. Most respondents assessed the maturity of both aspects to be on a “defined” level, which means that the process was identified in the organization, and roles and procedures were established, but improvement and support were required. A relatively high number of respondents assessed the processes in these areas at the “Repeatable process” level, and it should be remembered that none of the respondents assessed their projects to be low on the complexity or innovation scale. The implementation of larger initiatives, including innovative measures with only basic support provided by the organization concerning existing roles and processes, is a challenge which frequently generates a higher energy and cost demand. One person described the level of project management in their organization as fully optimized, whereas in the case of risk management, one person gave an extremely low assessment of “Awareness of process”.

## 4. Results

Due to the fact that a small research sample was included in the study, the numerical data presented below are not used for statistical inference. In the case of tables and figures, our aim is to present the distribution of responses to maintain clarity and transparency rather than to draw statistical conclusions. The data below are used to generate meaning from qualitative data, to document, verify and test interpretations, and to represent experiences [100].

### 4.1. The Pandemic As a Materialized Risk. The Preparation of Healthcare Organizations for Risk Management in Projects

An assumption can be made that the outbreak of the pandemic itself was an unprecedented event for the implementation of modern projects, and therefore it was difficult to anticipate and strategically prepare for. The first months of the pandemic afflicted the respondents with effects such as increase in material prices, unavailability of personal protective equipment (used as part of various projects performed under laboratory conditions), disruption of supply streams (import quarantine, delays in deliveries) and staff shortages (leaves and absences at work caused by quarantine).

Each of these and a significant portion of other phenomena that resulted from the pandemic may have been identified during the project’s risk analysis. Almost half of the respondents declared that in their organizations risk is analyzed as standard both before and during the implementation of all projects (in accordance with the guidelines of the selected methodology). They confirmed that the problems and challenges from the initial stages of the pandemic were identified and described earlier within the organization, which enabled a rapid and effective reaction after they materialized during the pandemic. Those managers expressed general satisfaction with the manner of dealing with pandemic-related risks, however a voice expressing clear objection could also be heard, indicating that the analysis conducted earlier in no way reflected the scale of later events. One of the participants said: “*Risks are usually specified in a general manner, and those pandemic-related ones which usually apply to human resources are the most difficult. I do not know anyone who would identify risk at the level of each team member concerning their temporary unavailability. And in the same manner, while the increase of the cost of goods and services could have been planned (with an anticipated reaction in the form of, for example, the increase of a particular cost), the materialization of so many expensive challenges was an unbearable burden for many projects*”.

In contrast, a considerate number of study participants had a problem with giving an unequivocal response to this question, indicating that although risk analysis is conducted, it is only for selected, strategic projects, or the analysis is, in their opinion, superficial and includes only the basic risks most frequently encountered during the projects. This is thus more of a way to meet the formal requirement related to the selection of a specific project management methodology, than a result of a conscious process of anticipating possible chances and problems (which corresponds to the results concerning the assessment of risk management maturity in the respondents’ organizations). A voice confirming that risk analysis in projects is not a standard action was also heard.

Finally, the participants emphasized that despite their efforts many aspects were beyond their reach and abilities. One of the study participants said: “*In my organization, dealing with pandemic-related risks consists mainly of extending the project performance period and reassigning funds within the project budget. This does not guarantee the full neutralization of effects, since budgets did not anticipate a risk with effects of such magnitude—e.g., the need to halt effective action for a year. Simultaneously institutions which subsidize projects do not anticipate any compensation (increases in financing) related to the pandemic*”.

### 4.2. The Assessment of the COVID-19 Pandemic’s Impact on the Criteria of Effectiveness of Implemented Projects

In the next part of the study, an assessment was made of the COVID-19 pandemic’s impact on individual dimensions of performance of the projects implemented in the studied organizations. For the assessment, six basic project management performance indicators used in the PRINCE2 methodology were used: time, cost, quality, scope, risk and benefits. The results are presented in Table 1.

All aspects of project implementation were affected negatively by the pandemic. The relatively least negative influence was observed in the aspect of benefits and quality, whereas the most negative impact concerned the area of risk and time.

The respondents jointly stated that the pandemic generated a significantly higher level of risk of not achieving the planned project results. In the comments, the frequently emphasized reasons included unavailable human resources (frequently the entire available forces were directed to fight the pandemic and provide medical services), in addition to the increase in costs or hindered communication in project teams.

An area which was also very strongly impacted is the management of projects’ time/schedule. The vast majority of implemented projects met with the need to extend the time needed for implementation in order to enable the achievement of the assumed results. As emphasized by one of the respondents “*The pandemic’s most significant impact was limiting the scope of activities in projects, which resulted in the need to extend them*”. Positive voices were also heard—in particular in the case of initiatives intended to handle the pandemic: “*A visible acceleration was related to the organization of call for projects. Suddenly it turned out that a call may be held, the applications evaluated, and the best project offers may be selected in just a few weeks. The market has shown us that it can adapt mostly to the accelerated operational tempo and we hope that these changes stay with us for longer, at least partially*”.

Another area negatively impacted by the pandemic was the management of project finances. One of the respondents emphasized the difficulties in fully estimating the increased costs of project implementation: “*While the costs of the tasks may be increased, it is not possible to calculate and settle the entire spectrum of additional costs related e.g., to the isolation or quarantine of important members of project teams*”. One of the project managers stated that the pandemic generally had a positive impact on the management of finances. Two reasons were indicated: first, releasing the purchase process from the public procurement law procedures, which enabled saving time and work of people, who would normally be engaged in preparing the documentation and conducting the tender. The second reason for such an assessment was the higher flexibility of sponsors and institutions financing projects, which enabled rationalizing the process of change management in the project. The pandemic has, to some extent, forced changes in the manner of performance of some initiatives (e.g., transforming the organization of training from a stationary to a remote mode), which resulted in measurable savings in some cases.

The last area in which the pandemic has mostly had a negative impact is the projects’ scope. The respondents emphasized that “*the pandemic has resulted in the need to stop substantive activities in the projects (e.g., seeing patients in clinical trials) while simultaneously continuing management and administrative processes (e.g., reporting, settlement). This results in the disturbance of correct proportions in the engagement of the organization’s resources*”. The increased risk of delays in the performance, and even of not achieving the assumed goals, was also emphasized. In the case of respondents who positively assessed the pandemic’s impact on the scope of the project, the more flexible procedures were mainly indicated, which enabled adapting the scope of implemented projects to the changing reality.

The last two areas subjected to analysis are “Benefits” and “Quality”. Half of the respondents indicated a negative or somewhat negative impact of the pandemic on achieving the planned benefits. When talking about benefits in healthcare projects, a greater focus is on social benefit and general welfare rather than the financial effect of the project. This is also, however, the only category where a positive impact of the pandemic was also indicated. The following reasons were given for such an assessment: acceleration of procedures in projects intended to prevent COVID-19 and the reduction in some costs related to specific types of projects. One of the respondents emphasized: “*Starting a clinical trial in our country usually takes at least half a year. Obtaining an approval of a trial by URPL (Office for Registration of Medicinal Products) may take up to 60 days. We are currently performing a few non-commercial clinical trials and the tempo in which all the requirements were met is astonishing. A few days after receiving information on financing we already had signed contracts for CRO services and for insurance. We only waited 2 days for URLP approval! Half a year of work was shortened to 5 days. This was, of course, only possible with the tremendous expenditure of work by each of the parties interested in the trial, but we all had a common goal—saving human lives*”.

In the case of quality, most respondents assessed that the pandemic had no impact. Among the persons which identified a negative impact, the most frequently appearing arguments concerned remote work: “*Initially we thought that this will all pass quickly and after a short hold-up of work we would be able to continue in previous conditions. It quickly turned out that our expectations were mistaken. In some projects we had to restart work. This lowered the quality*”. An argument was also made regarding the need to introduce remote education—which does not always allow the expected results to be achieved. The training of medical staff is frequently based on supporting group work ability and the practical use of the obtained theoretical knowledge. “A *significant problem in case of training programs during this time is the impossibility of predicting the attendance—whether the participants (medics) will not be assigned new duties at the very last moment or fall ill themselves. This means that it’s difficult to assess the possibility of training and they are frequently cancelled despite a huge interest*”.

### 4.3. Impact of the COVID-19 Pandemic on the Organization of Work of Project Teams

**Figure 3:**
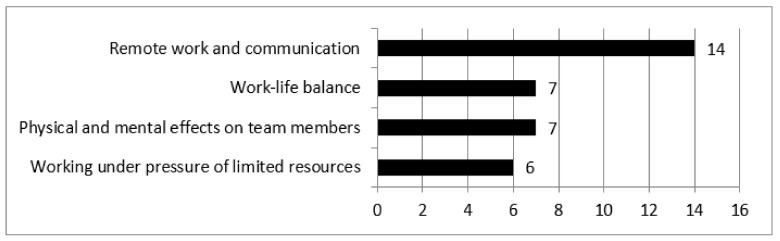
Impact of the COVID-19 pandemic on the organization of work of project teams.

In next part of the study, the impact of the COVID-19 pandemic on the organization of the work of the project teams in healthcare organizations was assessed. To this end, the respondents were asked about the most important changes and challenges. Based on the answers, 34 indications were identified, which were further divided into four groups, concerning: (1) changes in the area of communication and the introduction of remote work; (2) challenges in the area of work–life balance; (3) acting under pressure of limited resources; and (4) physical and mental impacts. The range of indications on individual areas of changes are presented in Figure 3.

The great majority of the respondent’s statements concerned changes in the area of communication in project teams: “*work which was required to be performed using the internal systems was performed by phone contact*” and organization of work in remote form. These changes were particularly onerous at the beginning of the pandemic, which mainly resulted mainly surprise, the very dynamic development of the situation, and restrictions on non-verbal and non-formal communication: “*communication in the team was the biggest challenge—we frequently ask each other about things when we are in the office. Suddenly this all was gone and solving simple problems started taking more time*”.

The barrier was then both “resistance by people we were working with”, insufficient competencies on part of the staff, e.g., “initial difficulties with mastering on-line teaching platforms”, and also technical problems: “with hardware at home, with the Internet”, “outdated software necessary for on-line meetings, no camera, loudspeakers etc”. One of the respondents pointed out the problem of compatibility between IT systems used in the organization and at home: “remote access to internal systems was difficult, since it was not possible to properly configure my laptop”. In these conditions the key role was played by the previous preparation of the IT infrastructure of healthcare organizations, which paid off at the moment of the outbreak of the pandemic. “At this time the investments made many years ago in remote access tools, communicators and network security devices have proven themselves”. Another voice was raised supporting the adaptation of other sectors’ technologies to healthcare requirements: “For many years I have been an advocate of digitization and the use of technological achievements, even if not directly related to healthcare. My ideas (e.g., telemedicine in chronic pulmonary diseases or cardiology) were rejected by sponsors (ministries, project financing agencies) as interesting but not crucial at the moment. Once I even heard: “Telemedicine? It won’t catch on!” I hope that, at least now, the weight of discussions in this aspect will change”.

The system of communication within project teams has changed significantly. One of the respondents emphasized that the main challenge was “*the lack of meetings. For me, this disrupted the flow of information and passing it on downward to the teams*”. Various solutions were used in order to ensure effective and efficient communication, all of which were fully remote: “*we mainly used social communicators (e.g., Messenger) where we created groups. Additionally, we met at on-line briefings*” or “*it was necessary to maintain group meetings at least once a week*”. External relationships between the team and the project environment also underwent changes. One of the respondents pointed out that there was a significant problem with “*anticipating the behaviors and attitudes of patients towards the need to contact medical personnel during the pandemic.* *Solution: interviews with the patients*”.

In another area, the respondents pointed to the challenges of work–life balance when working from home. Problems appeared in the first stage of the pandemic and included both the issues of space: “*finding a space at home which would enable concentrating solely on official duties*”, and technical issues. One of the respondents pointed out the difficulty of “*reconciling private matters, how the constraints impacted the organization of family life (in particular closed day care centers and kindergartens) with new professional duties (new COVID-19 related projects or team building—particularly difficult during remote/hybrid work)*”. Other managers emphasized the occurrence of new challenges in the area of project team monitoring: “*it was necessary to maintain team motivation for readiness and work in home environments. Due to various communication channels, including video conferences, it was possible to maintain good team morale*”, “*the lack of clear boundaries between the area of professional and private life of employees were additional difficulties. It was necessary to motivate employees to unequivocally separate these two areas and to take care to rest properly*”. Despite the occurring difficulties, the respondents have achieved successes in this area. One of them emphasized that “*the team has proven very reliable and has performed their task just like before the lock-downs were introduced. We did not have any pathological situations consisting of declaring work and not performing it. Only in one case out of less than twenty employees in my team was there a decrease of quality and the number of performed tasks*”.

The area of organization of work of project teams was acting under the significant pressure of limited resources. During the first stage of the pandemic “*the biggest problem was access to personal protective equipment*”. These problems were increased both by the lack of supplies and the increasing prices of the supplied resources: “*supply chains basically stopped working, even if goods were available in offers, their actual availability was illusory, and in light of significant demand the prices have grown disproportionately.*”

Second, staffing shortages made themselves known: “*availability of appropriate staff is a problem and I have not managed to deal with this till now*”. Available resources also decreased as a result of many additional tasks faced to healthcare organizations in conjunction with the outbreak of the pandemic: “*The COVID-19 pandemic has provided me with many new, previously unmet challenges*,” the “*additional, urgent performance of COVID-19 projects*” appeared. As a result, significant restrictions on time occurred, hindering “*keeping the schedules*” and resulting in the drop in other project performance criteria.

The aforementioned changes in the organization of work (remote work, pressure of limited resources and challenges of work–life balance) also resulted in many specific physical and mental impacts on project team members. First, the respondents indicated “*isolation, change in the personal and professional life,*” “*no contact with humans, with an adverse impact on the psyche*,” and “*no possibility of ‘working off’ the tension*”, which transforms into “*stress, which accompanies us until now*”. Work overload was also pointed out: “*overload with remote meetings. The ease of their organization and the possibility of switching between the meetings basically non-stop practically gives no time for regeneration, which during office work occurs (the need to move from one room to another or to travel to another location). I have shortened my participation in meetings where possible or I have not participated where it was not necessary*”, “*the need to work in the evenings (e.g., due to the need to share the space at home with other inhabitants). This probably resulted in the feeling of being continuously present at work, and thus higher mental fatigue, symptoms similar to overwork syndrome*”.

The negative impact of external information on the functioning of project teams was also a challenge for the management: “It seems that the highest challenge was to maintain a positive attitude with the simultaneous flood of purely negative information in the media. Although a few persons left work, we managed to transform our stress into a motivational element: we are here because we are needed, this is also our time of trial”. It seems that this type of thought may positively impact their managerial competences, ensuring higher efficiency and effectiveness of managing projects in healthcare organizations in the future.

Additionally, in the study, the respondents were asked to assess (positively or negatively) the aforementioned changes in the organization of the work of project teams. The results indicate that they identify both positive and negative effects to a very similar degree. Among the positives, the tighter focus on the most important project activities and the improvement of organizational procedures through their virtualization were the main factors listed. Negative effects such as remote work, higher workload and fatigue among employees, in addition to troubles with work–life balance, were listed as drawbacks.

### 4.4. The Impact of the COVID-19 Pandemic the Future of Project Management in Healthcare Organizations

In the last part of the study, the respondents were asked about the impact of the COVID-19 pandemic on the future in the sphere of project management in healthcare organizations. A vast majority of managers stated that pandemic experiences will change project management in the future. Moreover, additional comments were formulated, based on which 16 indications were identified, divided into three substantive groups, concerning: (1) higher flexibility in project management; (2) increased importance of risk management in projects; and (3) larger scope for the use of digital solutions in project management.

Most indications concerned the increase in digitization in project management in the future. Regarding this aspect, the pandemic was treated by the respondents as an opportunity for the introduction of new solutions, mainly concerning the digitization of organizational procedures, of document workflow and of remote work: “*paradoxically, the difficult situation has shown us new possibilities for management*”, “*the possibility to work remotely was created, which was previously not possible at my hospital. The human resources were so rigid, that there was only the option of coming in to work sick, or taking sick leave and staying at home. Some project tasks could have been effectively performed in such situations at home—without exposing others to possible infection*”. Similarly, according to another respondent, the pandemic will change the future of project management “*due to the need to introduce technology into project life.* *Most meetings, consultations are conducted on-line. The project is also managed remotely. This opens new possibilities*”. In accordance to the respondents in the future “*various types of improvements at the IT level will be used,” “there is a chance for the more frequent use of remote forms of training*”.

Digital changes will occur “in the area of communication between contractors and of interaction with stakeholders” of the projects. According to one of the respondents “it will bring more positive than negative effects—despite the lack of physical meetings with project partners the time needed for transport is saved, which will enable the intensification of other project activities”. The progressing digitization of project management will allow optimization of the length of project meetings, “increased effectiveness, better organization. However, it will negatively impact human relationships and team building, in my opinion”.

Another area identified by respondents concerns the impact of the COVID-19 pandemic on the increased importance of risk management in projects. This results mainly from the fact that “*the pandemic has made us all aware that even the best planned and organized tasks may be sorely tested in their effective implementation. The planned goals and assumptions very frequently could not have been carried out due to external factors (lockdown), despite willingness on the part of the implementing parties*”. As an effect in the future “*more attention will be paid to risk analysis,*” and “*all areas of risks which were not defined will become common for projects*”. According to one of the respondents, maintaining positive trends of increasing the risk management maturity level in healthcare organizations will pose a challenge to project management: “*the pandemic made the impact of a force majeure event real. For some attentive managers this may be in impulse for a more serious approach to risk management. I doubt, however, that it will widely change the manner of thinking about risk. I expect that project managers will treat the pandemic as a one-off event and after it ends, they will “forget” about it in the context of project planning*”. As a result, the pandemic may become an important factor in the increase in professionalization in project management only for some managers of healthcare facilities.

Some respondents also pointed out that the pandemic will be a source of more agility and flexibility in project management: “*Yes, the pandemic will change the manner of project management.* *They will have to be more flexible*,” “*it will be necessary to perform tasks when it’s possible and not when it’s needed, because it may turn out that when they are needed it will no longer be possible*”. This flexibility will mainly include “*faster decisions without long consultations and task delegation*,” “*shortening of official deadlines for many things,*” and “*the simplification of procedures—it turns out that procedures may be simpler, most matters can be settled on-line*”.

## 5. Discussion

The conducted empirical research and the analysis of the collected material enabled us to answer the four posed research questions:

RQ1: Have risk analyses in project management in healthcare organizations enabled a rapid and effective reaction due to the risk that materialized during the COVID-19 pandemic?

The results indicate that appropriate preparation and earlier actions concerning project risk analysis in the studied organizations have enabled a faster and effective reaction during the COVID-19 pandemic. Representatives of the studied healthcare organizations, in which risk analysis is conducted as a standard part of the implemented projects, confirmed that the risks from the initial stages of the pandemic were identified and described earlier in the organization, which enabled a rapid and effective reaction after they materialized during the pandemic. As a result, these managers expressed a general satisfaction about the manner in which they handled the risks related to the pandemic. Those projects were thus demonstrated to be somewhat resilient to sudden change and crisis resulting from the pandemic. Although resilience was not the subject of this study, the results may suggest that project management systems applied in participants’ organizations proved to be somewhat resilient. This may be due to the experience of working in complex systems (as the analysis included project initiatives of general hospitals on at least the 2nd reference level, which means they already have some ability and experience in the application of adaptation strategies). Even if they do not refer directly to resilience, their experience forced them to develop specific standards of conduct, ultimately building the potential for effective management in crisis. Projects and organizations which treat risk analysis as a standard, obligatory procedure seem to fulfill—in terms of project management—at least two steps suggested by E. Hollnagel [54]: anticipation and monitoring. If they employed any knowledge management system (suggested by project management standards), they took a step towards fulfilling the fourth measure: learning.

RQ2: In what manner has the COVID-19 pandemic impacted project management performance in healthcare organizations?

The main indicators of project effectiveness (such as: risk, time, cost, scope, benefits and quality) in the studied organizations were, to a lesser or greater degree, affected by the negative impact of the pandemic. Relatively, the most negative impact concerned the area of risk and time/schedule of the implemented projects. The management of project finances and the scope of conducted projects were affected negatively to a somewhat lesser degree. In the case of the indicator of benefits, the same number of respondents indicated a negative impact as a positive or no impact. In most cases, the pandemic had no impact on the parameter of quality of the performed projects.

RQ3: How did the COVID-19 pandemic influence the organization of work of project teams in healthcare organizations?

The pandemic significantly modified the organization of work of project teams in the examined organizations. The respondents identified four key areas of this influence: (1) concerning the introduction of remote communication both within project teams and in relations with external stakeholders, and also the introduction of remote work in the organization; (2) new challenges in the area of work–life balance which appeared, related mainly with the organization of work at home, ensuring the compatibility of IT solutions and motivating the project team; (3) the need for the performance of projects under the pressure of limited physical, human and time resources; and (4) the appearance of specific physical and mental impacts on project team members, such as isolation, stress, work overload, and physical and mental over-exertion.

RQ4: How will the COVID-19 pandemic impact the future of project management in healthcare organizations?

Study respondents believe that the COVID-19 pandemic will significantly impact the future of project management in healthcare organizations, mainly through: (1) the increased scope and importance of remote work and of digitization of organizational procedures and document workflows; (2) the increased importance of risk management in projects; and (3) the increased agility and flexibility in project management. Moreover, the respondents indicated the significant durability of changes in the area of digitization and the increase in flexibility of project management. Doubts were voiced concerning the area of increased importance of project risk management. There were opinions that permanent change in this area will occur only in some project managers in healthcare organizations.

The obtained results therefore confirm the unanticipated, sudden, significant and multidimensional impact of the COVID-19 pandemic on the functioning of healthcare organizations [101,102]. However, existing studies have mainly concentrated on physical, mental and psychological impact of pandemic on healthcare workers [103,104,105,106,107,108,109,110]; safety of healthcare workers [111,112]; course and scope of use of routines medical processes and services [113,114]; and digital transformation of the provision of healthcare services [115,116]. The conducted research provides new, deeper knowledge about the impact of the COVID-19 pandemic on practices of project management in healthcare organizations.

A significant part of the obtained results is consistent with the experiences of organizations from other sectors of the economy (particularly commercial businesses). This mainly concerns the significant role of risk analysis in project management under crisis conditions [117,118], the negative impact of the COVID-19 pandemic on the performance of implemented projects [65,66,67,68] and the significant impact of the pandemic on the organization of work of project teams, particularly including the introduction of rules for remote work [74,78,119]. Regarding the anticipated changes in the area of project management, the results correspond directly with conclusions concerning the increased importance of risk management [86], and the progress of virtualization and digitization in project management in the future [89,90], in addition to H4.0 adaptation in terms of building resilience of healthcare organizations.

In addition, the results indicate particular specifics of the pandemic’s impact on practices in project management in healthcare organizations. The study findings are consistent with the conclusions reached by A. Fraczkiewicz-Wronka et al. [120], which stated that Polish hospitals with well-developed risk management practices are much better prepared to find appropriate answers to external threats. This was confirmed in project management under COVID-19 pandemic conditions.

The obtained results also confirmed conclusions concerning rapid technological innovations occurring under the pressure of the COVID-19 pandemic, which applied mainly to remote control, remote working, telemedicine and remote learning [121]. It was found that, in the studied organizations, the jump was sudden and produced many important effects for project management. The digitization of management, in particular for administrative procedures and document workflow, is beginning to take center stage. This may form an important factor for reducing bureaucracy in public organizations, in which overly formalized rules, regulations and processes are frequently time-consuming and ineffective, and generate various types of delays (e.g., due to the need for manual processing of documents), which do not directly serve functional goals [122]. The results indicate that the pandemic has acted as an industry “disruption”, freeing a large amount of time and energy for project teams and administrative employees through the digitization and automation of document workflow processes. Healthcare organizations needed to quickly adopt H4.0 principles in terms of virtualization of activity and remote work. The pandemic enabled general management in the aspect of data access, team leading, or knowledge and change management.

From a more general perspective, the obtained results indicate the need to increase the professionalization [123] and even the certification [124] of project management in healthcare organizations. To date, the professionalization of project management in healthcare organizations has proceeded slowly. Most of the respondents indicated that this is at a level of defined or repeatable processes (II and III on a 5-degree scale). When we connect this with the fact that the same respondents participate in complex and difficult projects, it can be clearly seen that the professionalization of management is insufficient compared to the complexity of implemented initiatives. This is all the more important because, as shown by research conducted by J.C. Albrecht and K. Pang [125], project complexity should be closely related to project management maturity. It is thus expected that, in some of the studied organizations, the pandemic was a factor that put the system off-balance and reduced project performance.

None of the respondents, however, indicated that the pandemic would permanently influence the increase in professionalization of project management, e.g., by more accurately defining the principles of the project budget or scope management. This conclusion is particularly unfavorable in light of the increasing requirements concerning the development of project management competencies of healthcare managers and employees [126,127].

## 6. Limitations

When analyzing the obtained results, the limitations of the conducted study should be taken into account [128]. These limitations mainly include the small sample size and the inability to generalize the results. The pandemic restrictions and the workload of project managers meant that it was difficult to reach them and to invite them to a time-consuming study. Another problem was the pandemic itself, which represents a time of enormous stress, and may have negatively impacted the readiness to participate in the study, and the answers provided. It would be interesting to conduct a similar study in the period of the “new normal” [129], to see how many of the positive and negative effects of the pandemic for project management remained in the practice of the functioning of healthcare organizations. Another limitation of the study was the issue of fear of judgement. Although the respondents were ensured of their anonymity, which was maintained during the process of the collection and analysis of data, fear of judgement is natural, and may appear in such situations, particularly when respondents were asked about negative experiences, difficulties, problems in management, etc. This may have resulted in caution in giving answers and in balancing the wording to look good in the researcher’s eyes. Another restriction is that the sample was selected only in organizations with high renown (due to the personal contacts of the researchers, which therefore provided easier access to study subjects and easier methods to build trust; this was important due to the circumstances and work overload of study participants). As a result, the results do not provide answers concerning managerial responses to the onset of the COVID-19 pandemic in smaller healthcare organizations, or in organizations with a lower level of renown in the industry.

## 7. Conclusions

The COVID-19 pandemic has the nature of a global scale disruption [130]. The conducted study indicates that it also significantly impacts the practices of project management in healthcare organizations, both positively (e.g., by increased digitization and automation of procedures in the bureaucratized structures of public organizations) and negatively (e.g., by limiting project performance, or by causing a significant work overload of the management staff). Its main scientific contribution is confirmation of the effectiveness of risk analysis in project management under crisis conditions, and the assessment of the pandemic’s impact on project performance and the organization of the work of project teams, in addition to establishing the perspectives of changes in the area of project management in healthcare organizations in the future. The obtained results of the research also enable formulation of the following afterthoughts:

1. Despite the challenge posed by the outbreak of the pandemic, healthcare organizations managed to continue the projects during that period. After the first shock, which threw the organizations off-balance, adaptation measures were adopted relatively quickly. This may be due to appropriate ad hoc managerial decisions that were in the unstable environment of the onset of the pandemic. Study participants intuitively engaged methods used in agile methodologies when necessity forced them to. Taking this into consideration, more attention needs to be paid to professionalization in this area, because waterfall, traditional project management methods may now play only a supportive role.
2. The occurrence of a pandemic and its effects on project management in health care also resulted in unexpected, positive changes or implementations. Statements appeared in the study noting fewer bureaucratic procedures and faster decision making, which enabled more flexible processes and a number of other changes facilitating the work of project managers. Due to the pandemic, managers were forced to implement and adopt new solutions, which, unexpectedly, did not result in negative consequences (e.g., access from home did not cause sudden leaks of sensitive data). Without the pressure of the pandemic, these changes would probably not have been implemented for many more years, or even never. Nonetheless, the disruptive effect of the pandemic exposed ineffectiveness in some aspects, which was then used to accelerate the implementation of inevitable changes.
3. There is a risk that similar events may occur in the future, and such events are, unfortunately, difficult to predict. Hence, the diagnosed potential of project managers should be the basis for further training in order to build resilience and enhance adaptability, dynamic risk management and agile-oriented project management. If healthcare organizations find themselves in the new reality and are able to manage this environment, they will reach a completely new level of maturity, called “anti-fragility” by the contemporary thinker N. Taleb [131].

The importance of the obtained results for the improvement of project management practices in healthcare organizations simultaneously indicates the need to continue research. Concerning future, prospective directions of research, questions regarding the long-term impact of the pandemic on project management remain open. Therefore, analyses of the degree to which the consequences of the pandemic will be of a permanent nature, and how many of these will become only temporary solutions, will be particularly interesting. The analyses should also be expanded by identification and assessment of other, permanent effects of the pandemic’s impact on project management, which are currently not predicted.

Future research should also be directed to the identification and assessment of differences in the perception of the pandemic and its impact on project management in healthcare organizations of various sizes, complexities and industry prestige levels, and operating in various countries. This will enable the analysis of the extent to which structural and environmental variables (e.g., operating in different political and legal systems) impact handling such a significant and global risk as the COVID-19 pandemic.

Due to the relatively low professionalization of project management in Polish healthcare organizations, analyses of whether the chances resulting from the COVID-19 pandemic were taken advantage of will be important. These changes include, among others, the performance of many new projects related to infectious diseases, cooperation with various research and development entities from across the world, or access to medical data from international databases. Making use of these possibilities will provide an opportunity to increase the professionalization of project management and to make use of successful healthcare projects for a better socio-economic future in Poland.

## Figures and Tables

**Table 1:** The assessment of the COVID-19 pandemic’s impact on individual dimensions of effectiveness of implemented projects.

Project Management Performance Indicators	Negative Impact		No Impact	Positive Impact	
	Definitely	Somewhat		Somewhat	Definitely
Risk	7	13	0	0	0
Time	9	10	1	0	0
Cost	1	15	3	1	0
Scope	3	12	4	0	1
Benefits	1	9	7	3	0
Quality	1	5	14	0	0

## Data Availability

The data presented in this study are available on request from the corresponding author. The data are not publicly available due to privacy and ethical restrictions.

## Appendix A. Interview Protocol

*Part I—Respondent’s Data:*

1. Gender:
  − Female
  − Male
2. Your function in the performed projects:
  − Manager/Coordinator/Principal Investigator
  − Supervisor/Administrative coordinator/Functional manager
  − Representative of the board of directors supervising the performance of key projects
3. In what projects are you engaged in (multiple-choice question):
  − Educational and training
  − Preventive healthcare, e.g., the performance of a preventive study or another initiative of that type
  − Scientific and research
  − Information technology, e.g., consisting of software implementation
  − Infrastructural, e.g., consisting of the performance of construction and/or hardware investments
  − Clinical trials (also non-commercial)
  − General organization, e.g., projects of the management board intended to introduce new solutions in the organization
4. In what projects are you mainly engaged?
  − “Hard”—object-oriented, e.g., infrastructural projects
  − “Soft”—process-oriented, e.g., organizational change or training projects
  − “Hard” and “soft” to an equal extent

*Part II—General Characteristics of Project Management in the Organization:*

5. What is the operational range of the organization you are working at?
  − National
  − Regional
  − International
6. How do you assess the project management maturity level in your organization? (In the question each level was characterized, in order to increase the reliability of the respondent’s answer)
  − Awareness of process
  − Repeatable process
  − Defined process
  − Managed process
  − Optimized process
7. How do you assess the risk management maturity level in your organization? (In the question each level was characterized, in order to increase the reliability of the respondent’s answer)
  − Awareness of process
  − Repeatable process
  − Defined process
  − Managed process
  − Optimized process
8. How do you assess the degree of difficulty/complexity of the projects you have implemented: (in the question each level was characterized, in order to increase the reliability of the respondent’s answer)
  − Very high
  − High
  − Average
  − Low
  − Very low
9. What is the degree of innovation of the projects you are engaged in? (In the question each level was characterized, in order to increase the reliability of the respondent’s answer)
  − Very high
  − High
  − Average
  − Low
  − Very low

*Part III—Project Management During the COVID-19 Pandemic:*

10. How do you assess the preparation of your organization for the COVID-19 pandemic from the point of view of risk management in projects?
  **Question**	**I Agree**		**Not sure**	**I Do Not Agree**	
  	**Definitely**	**Somewhat**		**Somewhat**	**Definitely**
  In my organization risk analysis is conducted as standard as part of the implemented projects					
  The risks related to the pandemic were identified before they occurred					
  The earlier identification of risks enabled/would enable a rapid and effective reaction after they occurred during the pandemic					
  In general, I am satisfied with the manner in which we dealt with risks related to the pandemic					

Additional comment:

11. To what degree did the COVID-19 pandemic impact the level of effectiveness indicators of projects commenced earlier:
  **Project Management Performance Indicators**	**Negative Impact**		**No Impact**	**Positive Impact**	
  	**Definitely**	**Somewhat**		**Somewhat**	**Definitely**
  Risk					
  Time					
  Cost					
  Scope					
  Benefits					
  Quality					

Additional comment:

12. What were the most important changes and challenges in the area of the organization of work of project team during the COVID-19 pandemic for you?
13. Did the changes to the organization of the work of project team during the COVID-19 pandemic have positive results? If possible, provide examples.
  − Yes
  − No

Additional comment:

14. Did the changes to the organization of the work of project team during the COVID-19 pandemic have negative results? If possible, provide examples.
  − Yes
  − No

Additional comment:

15. Do you think that the COVID-19 pandemic will change the manner of project management in healthcare organizations in the future?
  − Yes
  − No/hard to say

Additional comment:
